# The Wall Eclipsing Sign on Pulmonary Artery Computed Tomography Angiography Is Pathognomonic for Pulmonary Artery Sarcoma

**DOI:** 10.1371/journal.pone.0083200

**Published:** 2013-12-31

**Authors:** Hui-Li Gan, Jian-Qun Zhang, Xiao-Yong Huang, Wei Yu

**Affiliations:** 1 Department of Cardiac Surgery, Beijing Anzhen Hospital, Capital Medical University, Beijing Institute of Heart, Lung and Blood Vessel Diseases, Beijing, China; 2 Department of Image Diagnosing, Beijing Anzhen Hospital, Capital Medical University, Beijing Institute of Heart, Lung and Blood Vessel Diseases, Beijing, China; Indiana University, United States of America

## Abstract

**Background:**

The objective of this study was to evaluate the imaging characteristics of pulmonary artery sarcoma (PAS) on pulmonary artery computed tomography angiography (PACTA) that can be used to differentiate between PAS and pulmonary thromboembolic diseases, including chronic thromboembolic pulmonary hypertension (CTEPH) and acute pulmonary embolism (APE).

**Methods:**

The clinical data and imaging characteristics of 12 patients with PAS, 156 patients with CTEPH, and 426 patients with APE who were treated at Beijing Anzhen Hospital from January 2007 to August 2013 were retrospectively analyzed. All patients underwent PACTA before treatment, and the diagnoses of PAS and CTEPH were all confirmed by surgical biopsy.

**Results:**

All 12 PAS patients were initially misdiagnosed and received inappropriate thrombolytic and/or anticoagulant therapy before they were referred for surgical intervention. The mean time from PACTA to surgical intervention was 5.5±3.7 months (range 2–11 months). On PACTA, the PAS lesion always eclipsed the wall of the pulmonary artery before infiltrating outside the pulmonary artery, which was termed the wall eclipsing sign. This sign was observed in all PAS patients but was not observed in any CTEPH or APE patients.

**Conclusions:**

PAS is a rare neoplasm with a poor prognosis, and is easily misdiagnosed as thromboembolic disease. The wall eclipsing sign on PACTA is pathognomonic for PAS, and patients with this sign should be investigated to confirm the diagnosis and should undergo surgical intervention as soon as possible, rather than receiving thrombolytic or anticoagulant therapy.

## Introduction

Pulmonary artery sarcoma (PAS) is an extremely rare neoplasm that is usually indistinguishable from acute or chronic thromboembolic disease of the pulmonary arteries on clinical and radiological findings. Acute pulmonary embolism (APE) can be cured by thrombolytic and/or anticoagulant therapy, and chronic thromboembolic pulmonary hypertension (CTEPH) is a severe condition that can potentially be cured by pulmonary thromboendarterectomy. PAS is usually incurable and has a very poor prognosis, and early diagnosis with radical surgical resection offers PAS patients the only chance of survival [1], [2]. However, the clinical manifestations of PAS are non-specific and very similar to those of thromboembolic disease, resulting in frequent delays in making the correct diagnosis and initiating proper treatment.

Although the incidence of PAS is very low, this disease should be included in the differential diagnosis of pulmonary thromboembolism, especially in patients who do not respond to thrombolytic/anticoagulant therapy or who present with no identifiable source for thromboembolic events [3]. The diagnosis of PAS is often missed or delayed for months or years. It is speculated that in the majority of patients with fast-growing pulmonary artery tumors and progressive cardiopulmonary dysfunction, the diagnosis is not established before death. To plan appropriate treatment, PAS should be suspected whenever there are specific clinical and radiological manifestations that can differentiate it from thromboembolic disease.

In our experience of differentiating between PAS and thromboembolic disease, we found that a specific characteristic observed on pulmonary artery computed tomography angiography (PACTA), which we termed the wall eclipsing sign, was pathognomonic for PAS. To evaluate the diagnostic value of this sign for differentiating between different types of pulmonary artery lesions, we retrospectively reviewed all the PACTA examinations performed in patients with pulmonary thromboembolic disease and PAS at Beijing Anzhen Hospital from January 2007 to August 2013.

## Methods

The Ethics Committee of Beijing Anzhen Hospital approved this retrospective study and waived the need to obtain patient consent for inclusion. Written informed consent for treatment was obtained from each patient prior to their surgical procedure or thrombolytic treatment.

Between January 2007 and August 2013, 168 patients admitted into Beijing Anzhen Hospital underwent pulmonary thromboendarterectomy for pulmonary thromboembolic disease. Pathological examination of the surgical biopsy specimens showed PAS in 12 patients and CTEPH in the other 156 patients. During the same period, 426 patients with acute pulmonary embolism (APE) received thrombolytic and/or anticoagulant therapy, and the pulmonary artery thromboembolic load decreased enough after treatment in all these patients to confirm the diagnosis.

All patients underwent chest radiography, lung perfusion scintigraphy, echocardiography, and PACTA before surgical intervention or thrombolytic/anticoagulant therapy. All patients were also screened for deep venous thrombosis (DVT) by dual color Doppler ultrasound examination. Unusual PACTA findings and a lack of response to thrombolytic/anticoagulant therapy led to a clinical suspicion of PAS in three patients, all of whom underwent positron-emission tomography (PET)-computed tomography (CT); two of these patients also underwent magnetic resonance imaging (MRI) of the chest.

The medical records of all patients were retrospectively reviewed to determine the clinical characteristics, therapeutic findings, operative findings, postoperative course, and long-term outcomes. The clinical characteristics of patients with PAS, CTEPH, and PAS are shown in Table 1. The PACTA findings were retrospectively analyzed to determine the specific imaging characteristics that differentiated PAS from thromboembolic diseases.

**Table 1:** Clinical characteristics of patients with APE, CTEPH, and PAS.

Group	APE	CTEPH	PAS	P value@
Case number, n	426	156	12	
Sex (male), n (%)	239(56.1)	91(58.3)	6(50)	0.7995
Age, yrs	47.1±14.6	48.9±13.8	45.4±8.8	0.3539
Normal D-dimer, n (%)	0(0)	94(65)	12(100)	0.000
DVT, n (%)	426(100)	92/156(65)	3/12(25)	0.000
Well score	7.6±1.81	4.6±2.47	1.39±1.21	0.000
Revised Geneva score	13.1±2.12	7.3±3.55	4.59±1.90	0.000
Abnormal hs-CRP, n (%)	27(6.3)	12(7.7)	12(100)	0.000
Abnormal BNP, n (%)	421(98.8)	156(100)	8(66.7)	0.000
Abnormal ESR, n (%)	25(5.9)	13(8.3)	9(75)	0.000
Abnormal LDH, n (%)	11(2.6)	8(5.1)	8(66.7)	0.000
duration of symptoms	10.3±8.7d	32.2±26.4 m	14.6±12.8 m	0.000
Syncope, n (%)	267(63.7)	87(55.7)	9(75)	0.2048
Cough, n (%)	289(67.8)	96(61.5)	8(66.7)	0.3630
Dyspnoea, n (%)	426(100)	156(100)	12(100)	1
Hemoptysis, n (%)	124(29.1)	42(26.9)	3(25)	0.8439
Chest pain, n (%)	103(24.2)	31(19.9)	2(16.7)	0.4798
6MWD, m	—	326±89	289±76	0.163*
WHO function class (III+IV), n (%)	426(100)	156(100)	12(100)	1

DVT: deep vein thrombosis; hs-CRP: high-sensitivity C-reactive protein; BNP: B-type natriuretic peptide; ESR: erythrocyte sedimentation rate; LDH: lactate dehydrogenase; 6MWD: 6-minute walk distance.

@ Comparison among patients with APE, CTEPH, and PAS.

*Comparison between patients with CTEPH and PAS.

PACTA was performed using a 320-row multi-detector CT scanner (Aquilion ONE; Toshiba Medical Systems, Otawara, Japan) with 0.5-mm detector elements, 350 ms gantry rotation time, and scanner settings of 120 kV and 350–450 mA depending on patient anatomy. The scans included the entire pulmonary area. Patients were injected with 50 ml of contrast medium with 350 mg of iodine/ml at 4 ml/s, followed by 30 ml of saline solution. The automatic sure-start option of the CT scanner was used to achieve adequate contrast enhancement in the pulmonary artery. All examinations were performed according to the normal routine for diagnosis or evaluation of CTEPH or APE.

The 12 patients with PAS all underwent standard pulmonary thromboendarterectomy under general anesthesia with deep hypothermic circulatory arrest. The operation was performed through a median sternotomy. Embolectomy and endarterectomy were performed according to standard procedures, and further distal embolectomy was also performed to evacuate superimposed thrombosis and metastasized PAS embolus in the distal segmental pulmonary arteries. In patients with PAS extending from the pulmonary valve to the pulmonary trunk, the tumor was carefully peeled off the valve and arteries without valve replacement or graft replacement of the pulmonary artery. The main part of the PAS could be fully resected in all patients. The mean cardiopulmonary bypass time was 198.5±37.6 min, the mean aortic clamping time was 93.2±23.8 min, and the mean circulatory arrest time was 36.5±13.6 min. Postoperative adjuvant treatment included radiation therapy in three patients, chemotherapy in two patients, and combined radiation and chemotherapy in four patients. Three patients did not receive any adjuvant treatment.

The 156 patients with CTEPH all underwent standard pulmonary thromboendarterectomy under general anesthesia with cardiopulmonary bypass and deep hypothermic circulatory arrest. The operation was performed through a median sternotomy. The mean cardiopulmonary bypass time was 236.6±34.2 min, the mean aortic clamping time was 91.6±22.7 min, and the mean circulatory arrest time was 36.8±13.7 min.

The 426 patients with APE all received thrombolytic therapy with recombinant tissue plasminogen activator (50–100 mg) or urokinase (1–1.5 million IU) administered intravenously over 2 h, followed by heparin therapy.

The therapeutic regimens during the follow-up period varied, and included administration of diuretics, cardiac glycosides, and calcium channel blockers (diltiazem) to lower pulmonary artery pressure. Some patients with residual pulmonary hypertension received selective pulmonary vasodilator therapy including prostanoids, endothelin receptor antagonists, and PDE5-inhibitors. All the surviving patients were treated with warfarin, with the international normalized ratio (INR) maintained between 2.0 and 3.0.

Complete follow-up information was obtained for all 12 patients with PAS, 152 patients with CTEPH, and 421 patients with APE by mail, email, or telephone interviews or from outpatient department records. All patients underwent PACTA, MRI, and echocardiography at 3 months after thromboendarterectomy or thrombolytic therapy, and yearly thereafter. Follow-up data for the surviving patients were recorded until July 2013.

All statistical analyses were performed using SAS for Windows, version 8.2 (SAS, Cary, NC). Categorical data are presented as numbers and frequencies. Continuous data are presented as mean ± standard deviation. Patients with PAS, CTEPH, and APE were compared using the log-rank test. A value of p<0.05 was considered statistically significant.

## Results

### Diagnostic data

The patients with PAS were six males and six females with a mean age of 45.4±8.8 years. The presenting symptoms included syncope (n = 9), cough (n = 8), dyspnea (n = 12), hemoptysis (n = 3), and chest pain (n = 2). In patients with PAS, the mean lactate dehydrogenase (LDH) level, high-sensitivity C-reactive protein (hs-CRP) level, B-type natriuretic peptide (BNP) level, and erythrocyte sedimentation rate (ESR) were all elevated, but the mean D-Dimer level was in the normal range. In patients with CTEPH and APE, the mean LDH level, hs-CRP level, and ESR were in the normal range, but the mean BNP level was elevated.

In patients with PAS, the mean Wells score was 1.39±1.21 and the mean revised Geneva score was 4.59±1.90, indicating a low probability of acute pulmonary thromboembolic disease. Two of the 12 patients with PAS had DVT. All 12 patients received thrombolytic/anticoagulant therapy before they were referred for surgical intervention. None of these patients responded to thrombolytic/anticoagulant therapy, and one patient developed severe heparin-induced thrombocytopenia after long-term heparin treatment. The mean time from detection of a pulmonary lesion to surgical intervention was 5.5±3.7 months (range 2–11 months).

### Early results of therapy

Before referral for surgical intervention, the patients with PAS received a mean number of 1.45±0.32 thrombolytic treatments and a mean duration of 5.5±3.7 months (range 2–11 months) of anticoagulant therapy.

In patients with PAS, there were two in-hospital deaths after surgical intervention (2/12, 16.7%). One patient died from perioperative heparin-induced thrombocytopenia after a long duration of heparin therapy before referral for surgical treatment, and the other patient died from severe reperfusion pulmonary edema. In patients with CTEPH, there were four in-hospital deaths after surgical intervention (4/156, 2.6%). In patients with APE, there were three in-hospital deaths (0.7%) due to severe brain hemorrhage after thrombolytic therapy, and 426 patients responded well to the thrombolytic/anticoagulant therapy.

### Pathological examination findings in patients with PAS and CTEPH

In all 12 patients with PAS, the main part of the PAS lesion was fully resected and tumor embolus and thromboembolism distal to the tumor were completely evacuated. The tumors were gray-white, firm, gelatinous, polypoidal masses, measuring 6–10 cm in length and 3–5 cm in width. In 10 patients, the tumor was shaped like the pulmonary trunk and extended to the segmental pulmonary arteries, adhering to the arterial wall and the pulmonary valve. All the tumors were diagnosed and histologically classified by pathology specialists at Beijing Anzhen Hospital. Immunohistochemical staining was performed with antibodies to muscular actin, vimentin, h-caldesmon, CD34, CD117, S-100, smooth muscle actin, desmin, and CD68 according to previously described protocols [4], [5]. The histological and immunohistochemical findings of the patients with PAS are shown in Table 2. Pathological examination confirmed the diagnosis in all 156 patients with CTEPH.

**Table 2:** Clinical characteristics and pathological findings in patients with PAS.

Patient No.	Age/Sex	Initial diagnosis	Histological subclassification	Size(cm) length*diameter	Oringin of tumor
1	35/F	CTEPH	Leiomyosarcoma	8*5	Right pulmonary Ar.
2	41/M	CTEPH	Leiomyosarcoma	11*9	Pulmonary trunk
3	39/F	CTEPH	Undifferentiated sarcomas	7*4.5	Pulmonary trunk
4	43/M	CTEPH	Pleomorphic rhabdosardoma	8*4.5	Pulmonary trunk
5	54/F	CTEPH	Intimal sarcoma	7*5	Pulmonary trunk
6	52/F	CTEPH	Intimal sarcoma	10*4.5	Pulmonary trunk
7	38/M	CTEPH	Leiomyosarcoma	10*6	Pulmonary trunk
8	50/F	CTEPH	Leiomyosarcoma	9*3.5	Right pulmonary Ar.
9	47/M	CTEPH	Intimal sarcoma	6*3	Pulmonary trunk
10	36/M	CTEPH or PAS	Intimal sarcoma	8.5*4.5	Pulmonary trunk
11	43/M	CTEPH or PAS	Leiomyosarcoma	5*3	Pulmonary trunk
12	67/F	CTEPH or PAS	Intimal sarcoma	9*4.5	Pulmonary trunk

F: female; M: male; CTEPH: chronic thromboembolic pulmonary hypertension; PAS: pulmonary artery sarcoma; Ar: artery.

### Imaging examination findings

In all patients with PAS, chest radiography showed enlargement of the pulmonary trunk, left pulmonary artery, or right pulmonary artery; and echocardiography showed right ventricular dilatation, tricuspid regurgitation, and absence of blood flow into the pulmonary artery. PACTA showed intraluminal defects in the pulmonary trunk, left pulmonary artery, or right pulmonary artery, extending to the segmental arteries which had local aneurysmal dilatations. Ten patients (10/12, 83.3%) had a filing defect of the main pulmonary artery, 10 patients (10/12, 83.3%) had a filling defect of the left pulmonary artery, and 12 patients (12/12, 100%) had a filling defect of the right pulmonary artery.

**Figure 1:**
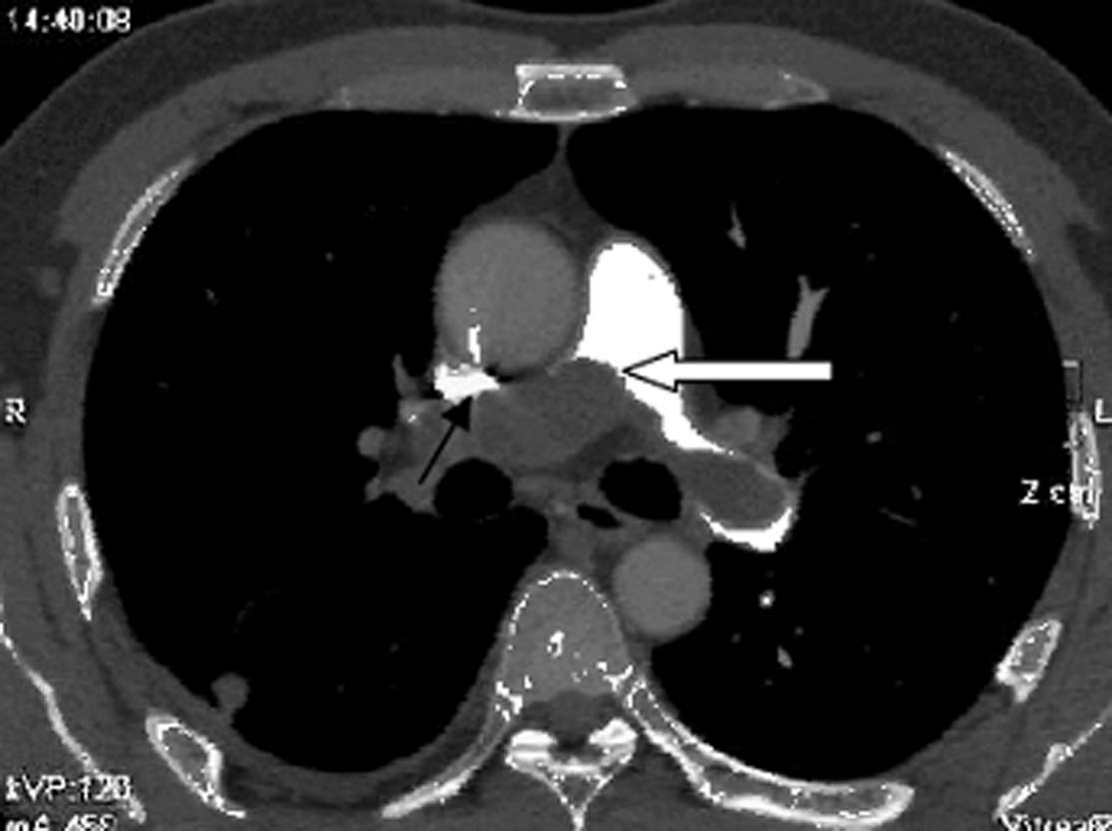
Pulmonary artery computed tomography angiography, axial view. The lumen of the pulmonary trunk is obliterated by a low-density mass (white arrow) that extends into the left and right pulmonary arteries. Both walls of the left pulmonary artery are eclipsed by the lesion (black arrow). The pulmonary trunk, left pulmonary artery, and right pulmonary artery are fully or partially occupied by the lesion, and the proximal end of the lesion protrudes towards the right ventricular outflow tract (white arrow). We termed this appearance the wall eclipsing sign.

In all patients with PAS, PACTA showed eclipsing of the wall of the pulmonary artery before the lesion infiltrated beyond the artery, which we termed the wall eclipsing sign (Figure 1). The wall eclipsing sign was defined as presence of the following three findings: almost full occupation of the lumen of the pulmonary trunk, left pulmonary artery, or right pulmonary artery by a low-density mass; protrusion of the proximal end of this mass towards the right ventricular outflow tract; and eclipsing of one or both walls of the pulmonary trunk, left pulmonary artery, or right pulmonary artery by this lesion. The wall eclipsing sign was not observed in any of the patients with APE or CTEPH (Table 3).

**Table 3:** Distribution of the wall eclipsing sign according to diagnosis.

Group	Total cases	WES(−)	WES(+)
APE group	426	426	0
CTEPH group	156	156	0
PAS group	12	0	12
P value@	0.000		

WES (−): wall eclipsing sign negative; WES (+): wall eclipsing sign positive.

@ Comparison among patients with PAS, CTEPH, and APE.

## Discussion

Primary PAS was first described by Mandelstamm in 1923 [6], and is very rare, with fewer than 300 cases reported worldwide to date [7], [8]. The typical age of onset is 45–55 years (range 13–86 years) with a female to male ratio of 2∶1. The most commonly reported symptoms are dyspnea, cough, chest pain, and syncope; and patients may also have weight loss, fever, or anemia [9]. In our series, retrospective analysis of the symptoms at presentation showed similar findings. The mean age at presentation was 45.4±8.8 years (range 35–67 years) and the series included six males and six females. Most patients presented with syncope, cough, dyspnea, and right heart failure, and some also complained of hemoptysis and chest pain. These symptoms are all characteristic of significant pulmonary vascular obstruction, and they are similar to the symptoms of thromboembolic disease, leading to misdiagnosis.

PAS is usually detected at an advanced stage, making curative resection nearly impossible [14]. The prognosis of PAS is therefore extremely poor. The natural course of the disease is determined by local tumor growth as well as superimposed thrombosis and metastasized tumor embolus, leading to progressive obstruction of the pulmonary vessels [10], [11]. The median survival of time of untreated patients after diagnosis is 1.5 months (range 1.5–5.5 months) [12], [13]. The main cause of death is decompensated heart failure [9].

Because of the rarity of PAS, it is often initially misdiagnosed as acute or subacute massive pulmonary thromboembolism or CTEPH. Anderson et al. [14] reported a series of six cases of PAS, all of which were initially investigated for chronic thromboembolism. Parish et al. [15] reported nine cases of PAS over a 30-year period, of which seven were originally treated for pulmonary thromboembolism. Huo et al. [16] reviewed the reports of seven cases of PAS, of which five were initially thought to be chronic thromboembolism. We report here 12 cases of PAS, all of which were misdiagnosed as CTEPH before they were referred for surgical intervention. These data show that PAS should be included in the differential diagnosis of pulmonary thromboembolism.

Because of the similar clinical presentations, it is very difficult to differentiate between PAS and pulmonary thromboembolism, leading to inappropriate treatments such as thrombolysis and long-term anticoagulant therapy [17]–[20]. Such misdiagnosis and inappropriate treatment result in delays of several months before surgical intervention, increase the risk of morbidity, and reduce the survival time. In our series, patients with PAS received a mean number of 1.45±0.32 thrombolytic treatments and a mean duration of 5.5±3.7 months (range 2–11 months) of anticoagulant therapy. In spite of improvements in imaging modalities, the diagnosis of PAS is still based on pathological examination findings, and the majority of specimens are obtained at surgery or autopsy. Preoperative histological diagnosis is usually not possible, although a biopsy specimen can sometimes be obtained by CT-guided transthoracic aspiration, pulmonary angioscopy with transvenous catheter suction biopsy, or transbronchial biopsy [21].

If PAS is diagnosed early, cure may be possible with aggressive surgical intervention. However, the diagnosis is difficult and often delayed because the symptoms are insidious and nonspecific [9]. These characteristics suggest that clinicians should increase their awareness of this disease to increase the likelihood of early diagnosis and treatment. As PAS is rare, it has not been studied in large randomized trials, and the optimal methods of diagnosis and treatment are still unknown.

There is no specific biomarker to assist in screening for PAS or differentiating it from thromboemlic disease. In our series, the hs-CRP level was elevated in all patients with PAS, the LDH level was elevated in eight patients (8/12, 66.7%), and the ESR was elevated in nine patients (9/12, 75%). In patients with thromboembolic disease the hs-CRP level, LDH level, and ESR were usually normal. These findings suggest that elevation of hs-CRP, LDH, and ESR in patients with a lesion in the pulmonary artery indicate PAS. Two of the patients with PAS had lower extremity DVT, indicating that the presence of DVT does not rule out PAS. The mean Wells score was 4.59±1.90 and the mean revised Geneva score was 1.39±1.21 in patients with PAS, indicating a low probability of acute pulmonary thromboembolism. These scores may help to differentiate between PAS and thromboembolic lesions.

As it is usually not possible to differentiate between PAS and acute or chronic thromboembolic disease based on clinical findings, patients are initially treated with anticoagulant therapy or even thrombolysis. No studies evaluating outcomes after long-term anticoagulant therapy in patients with PAS have been reported. However, many case reports suggest that failure of anticoagulant therapy to improve the patient's condition should raise the suspicion of PAS, and should lead to early consideration of biopsy or surgery to confirm the diagnosis, and early treatment to prolong survival.

Imaging examination findings are very useful for diagnosing PAS. Chest radiography findings are always nonspecific in PAS patients. Ventilation-perfusion scintigraphy is of limited value in differentiating PAS from thromboembolic disease. Several reports suggest that PACTA and MRI may be the most useful investigations for differentiating between tumor and thrombosis [22]. PACTA findings are similar in PAS and thromboembolic disease, and are characterized by filling defects in the pulmonary vessels. Enhancement of the lesion on gadolinium-MRI indicates a tumor, as a non-vascularized intraluminal thrombus does not enhance after injection of gadolinium. No information regarding enhancement in patients with sarcoid infiltration of the pulmonary artery was found in the literature; however, 80% of patients with active infiltrative lung disease have positive MRI findings.

The radiologist is usually the first to raise the suspicion of PAS in patients with severe dyspnea and pulmonary artery filling defects who are unresponsive to anticoagulant therapy. Combined CT and PET-CT findings are very useful for assessing patients with suspected PAS. Early diagnosis with the help of integrated imaging examination findings is the main factor required to obtain improvements in prognosis.

In this study, PACTA findings were useful for differentiating between PAS and pulmonary embolism. The characteristics suggestive of PAS include a filling defect occupying the entire lumen of the pulmonary trunk with an increase in diameter of the involved vessel and delayed patchy contrast enhancement, which is more evident in the venous phase. PET-CT was performed to differentiate between PAS and APE based on the intensity of contrast enhancement. MRI was also sometimes performed in patients with equivocal results on PET-CT, to improve tissue characterization of the lesions and differentiate between thromboembolism and neoplasm.

Based on preoperative imaging examination findings, nine patients were thought to have CTEPH, and only three were correctly diagnosed with PAS based on PET-CT findings. However, this retrospective study found that all 12 patients with PAS had the wall eclipsing sign, and that all patients with APE and CTEPH did not have this sign. We therefore consider the wall eclipsing sign on PACTA to be pathognomonic for PAS. When the wall eclipsing sign is observed, further investigations should be performed to confirm PAS, or the lesion should be resected. All the patients in our series were inappropriately treated with thrombolytic/anticoagulant therapy for a mean period of 5.5±3.7 months (range 2–11 months), and some of them suffered severe complications due to long-term anticoagulant therapy or thrombolysis such as gastric bleeding, heparin-induced thrombocytopenia, and liver injury. Such long delays before patients receive the correct treatment results in many patients losing the chance of survival. When the wall eclipsing sign is detected on PACTA, patients should undergo PET-CT to confirm the diagnosis, or should undergo surgical intervention as soon as possible, rather than receiving thrombolytic/anticoagulant therapy.

PAS frequently arises in the pulmonary trunk, and extends into the proximal and distal pulmonary arteries. PAS is classified into two types: intimal and intramural. Intimal PAS arises from totipotent intimal stem cells, and may be classified histologically as angiosarcoma (the most frequent), osteosarcoma, or rhabdomyosarcoma. Intramural PAS arises from the media of the vessel wall and is classified histologically as leiomyosarcoma [23], [24]. Both intimal and intramural PAS therefore arise from the wall of the pulmonary artery, resulting in eclipsing of at least one wall of the artery by the lesion. When the lesion grows large enough to invade the opposite wall of the pulmonary artery, both sides of the wall are eclipsed. In pulmonary thromboembolic disease, the lesion always originates in a deep vein of the lower extremity, embolizes, and lodges in the lumen of the pulmonary artery. Thromboembolic lesions therefore do not eclipse the arterial wall, and the wall eclipsing sign is pathognomonic for PAS. In late stage PAS, the tumor may invade structures adjacent to the wall of the pulmonary artery, or may metastasize to other important organs to an extent that cannot be surgically resected. In cases of invasion through the wall of the pulmonary artery, the wall eclipsing sign would no longer be observed.

## Conclusion

PAS is a rare neoplasm with a poor prognosis, and is easily misdiagnosed as thromboembolic disease. We consider that the wall eclipsing sign on PACTA is pathognomonic for PAS. When this sign is observed, the patient should undergo further investigation to confirm the diagnosis of PAS, or should undergo surgical intervention as soon as possible, rather than receiving thrombolytic/anticoagulant therapy.

This study is a retrospective analysis of very small series, and it is possible that there are confounding factors involved in the relationship between the wall eclipsing sign and PAS, and the results of this study are therefore very preliminary. More reports of cases or series of cases of PAS would reduce bias. The results of this study may help to guide the diagnosis and treatment of PAS.
